# HyperFace: A Deep Fusion Model for Hyperspectral Face Recognition

**DOI:** 10.3390/s24092785

**Published:** 2024-04-27

**Authors:** Wenlong Li, Xi Cen, Liaojun Pang, Zhicheng Cao

**Affiliations:** 1 Molecular and Neuroimaging Engineering Research Center of Ministry of Education, School of Life Science and Technology, Xidian University, Xi’an 710126, China; 2 School of Telecommunications Engineering, Xidian University, Xi’an 710126, China

**Keywords:** face recognition, hyperspectral, deep learning, image fusion, infrared

## Abstract

Face recognition has been well studied under visible light and infrared (IR) in both intra-spectral and cross-spectral cases. However, how to fuse different light bands for face recognition, i.e., hyperspectral face recognition, is still an open research problem, which has the advantages of richer information retention and all-weather functionality over single-band face recognition. Thus, in this research, we revisit the hyperspectral recognition problem and provide a deep learning-based approach. A new fusion model (named HyperFace) is proposed to address this problem. The proposed model features a pre-fusion scheme, a Siamese encoder with bi-scope residual dense learning, a feedback-style decoder, and a recognition-oriented composite loss function. Experiments demonstrate that our method yields a much higher recognition rate than face recognition using only visible light or IR data. Moreover, our fusion model is shown to be superior to other general-purpose image fusion methods that are either traditional or deep learning-based, including state-of-the-art methods, in terms of both image quality and recognition performance.

## 1. Introduction

Face recognition has been a topic of intense research for decades. Early research efforts mostly focused on designing robust feature extractors invariant to factors such as illumination, pose, expression, occlusion, blur, and external noise [1], while a certain level of practical success was achieved. Examples of well-known algorithms include Elastic Bunch Graph Matching (EBGM) [2], EigenFace [3], FisherFace [4], and Local Binary Patterns (LBPs) [5], to name a few. While much progress had been made by the early 2000s, most of these research works were limited to visible light. However, the performance of visible light face recognition algorithms degrades dramatically when tested in many real-world environments, for instance, at nighttime or under harsh atmospheric conditions. Such critical conditions are frequently encountered in civil, law enforcement, and military scenarios, such as public facility surveillance, border patrol, and battlefield intelligence.

More recently, face recognition in the infrared (IR) spectrum has become an area of interest due to its advantages over visible light face recognition [6,7]. IR is much less susceptible to lighting changes, which greatly alleviates the tricky issue of illuminance instability between enrollment and query data. Furthermore, compared to visible light images, thermal IR images retain a different type of information—the patterns associated with the heat emission of an object—which contain information about additional anatomical structures and are informative enough to distinguish between identical twins [8]. Last but not least, IR (especially short-wave IR) is used to sense the environment at night and under harsh atmospheric conditions such as rain, snow, and fog [9,10], making it a reliable imaging alternative to visible light in many real-world scenarios such as military and law enforcement applications. Dealing with different IR bands, by either stating the face recognition problem as an *intra-spectral* or a *cross-spectral* problem, has attracted the attention of several research groups [11,12,13,14,15,16,17,18,19,20,21,22,23,24,25,26,27]. Intra-spectral IR face recognition usually considers a single band within the IR spectral range and matches IR face images against other IR face images within the same band [12]. Cross-spectral face recognition [14,15,28], on the other hand, involves both IR and visible light and matches IR images to the visible light image gallery.

However, one noticeable drawback of IR face images is that compared to visible light images, they have a much lower resolution, and thus their textural information is not rich enough [29]. As a result, face recognition involving IR images usually has lower accuracy compared to face recognition with visible light alone. Thus, it has become clear that both modalities would benefit from their fusion [30,31], which, in this paper, we will refer to as *hyperspectral face recognition*. Hyperspectral face recognition typically involves fusing images acquired in different light subbands together, followed by applying a matching algorithm to the fused faces. Hyperspectral face recognition has the excellent characteristics of richer information retention and functionality under harsh atmospheric conditions. It is able to work under all weather conditions while maintaining a high recognition rate. Additionally, it resolves the previously mentioned issue of illuminance variance. Since hyperspectral sensors are already available, the focus of this work is on imaging fusion and recognition aspects rather than imaging and sensor aspects. More specifically, in this work, we involve two light bands (near IR and visible light) and try to fuse them together.

While hyperspectral face recognition has become and remained an active research topic [32], some problems remain unsolved. Firstly, most existing hyperspectral face recognition algorithms adopt traditional hand-crafted operators that clearly do not perform at the full potential of the hyperspectral data or usually have poor generality in different cases. Secondly, among the few deep learning-based methods for hyperspectral face fusion, many of them fail to consider the particularity of the hyperspectral context when designing the networks or models. For instance, the issues of local maximum and gradient vanishing are neglected, as the multispectral face dataset is much more limited in size compared to the visible light dataset. Lastly, many hyperspectral face recognition methods involve designing an image fusion algorithm, which usually yields a high-quality output of the fused face but does not necessarily guarantee high performance in face recognition. In other words, the image fusion algorithm needs to be specially designed and should be recognition-oriented.

Having observed the limitations of existing hyperspectral face recognition methods, in this paper, we draw on our experience in deep learning as well as on the abundance of deep learning toolsets available to us. We offer a new deep learning-based approach to hyperspectral face recognition. To the best of our knowledge, this work presents the first such effort. We propose a novel fusion model of deep neural networks that combines IR and visible light face images, named *HyperFace*. The DNN model is characterized by bi-scope residual dense learning, a feedback-style decoder, and a recognition-oriented loss function.

We make the following contributions to the scope of hyperspectral face recognition:

- We introduce deep learning to the problem of hyperspectral face recognition in order to overcome the shortcomings of traditional methods. To the best of our knowledge, this is the first time deep learning has been used to solve the problem of hyperspectral face recognition.
- A hyperspectral face fusion model is proposed, named HyperFace, which is built as a Siamese encoder–decoder, and it features a pre-fusion scheme.
- We propose bi-scope residual dense blocks with both local and global learning to enhance the feature representation ability of the network. A feedback connection is also introduced to further improve the reconstruction performance.
- We propose a composite loss function that comprises critical components such as structural loss, pixel loss, and facial detail loss. This composite loss is face recognition-oriented and enhances the final recognition accuracy.
- A complete hyperspectral recognition framework is provided, where transfer learning is involved at the recognition stage after hyperspectral face fusion. Experiments prove the superiority of our algorithm over other algorithms, including state-of-the-art methods, in terms of both fusion image quality and recognition rates.

The rest of this paper is organized as follows. In Section 2, we briefly review related works. In Section 3, the proposed hyperspectral face image fusion method based on deep learning is introduced in detail, and the proposed deep learning recognition network is described. Section 4 describes the datasets employed in this paper. The experimental results are presented and analyzed in Section 5. The conclusions of this work can be found in Section 6.

## 2. Related Works

The literature contains a considerable number of works on face recognition involving the IR band. However, most of these works either investigated the intra-spectral recognition case (i.e., IR vs. IR) or the cross-spectral recognition case (IR vs. visible light), leaving the hyperspectral recognition case understudied. There are only a handful of publications in which IR and visible light face images are fused and then used for recognition. We review several of these research works below.

Bebis and Pavlidis [30] were the first to introduce the concept of image fusion into face recognition for the purpose of performance boosting. They fused the infrared facial image and visible facial image in the wavelet domain. The genetic algorithm (GA) was used to select fusion information, and finally, a fused facial image of both light bands was obtained. Their experiment of fused face recognition proved that this method can effectively improve the accuracy of face recognition.

Since then, more fusion methods have been applied to face image fusion. For instance, Chen et al. [33] proposed a fusion method based on integral wavelet frame transform (nonseparable wavelet frame transform, NWFT) and Independent Component Analysis (ICA). The fusion recognition method combined the registered NWFT light image and infrared image fusion and used ICA to extract facial image-independent characteristics. A top-match method was used to classify the fused faces.

In their work [34], Abidi et al. fused visible and thermal IR faces for illumination-invariant face recognition, taking advantage of the fact that the thermal IR is insusceptible to light variance. The proposed multiscale data fusion method was based on discrete wavelet transform (DWT) and considered the issue of eyeglass replacement. Experiments demonstrated improved recognition accuracy under a wide range of illumination changes.

In 2008, Singh et al. [31] used the discrete wavelet transform (DWT) method combined with the Gabor transform to extract the amplitude and phase features of the fused image. They then used the adaptive SVM method to merge the extracted features, achieving a recognition effect with an error rate of 2.86%—the highest recognition accuracy at that time.

Ma et al. [35] designed a matching fraction fusion method based on two-dimensional linear discriminant analysis and applied Euclidean distance corresponding similarity to perform similarity fusion.

The above results show the advantages of hyperspectral face recognition (face fusion of different spectra) over a single spectrum and how hyperspectral face recognition can effectively improve face recognition accuracy. However, since earlier publications on hyperspectral face recognition involved hand-picked features, the optimality of hyperspectral face recognition remains an open research problem. In their review paper, Jain et al. [32] made a detailed comparison of existing traditional face image fusion algorithms and concluded that all traditional methods of hyperspectral face fusion suffer from issues such as manually designed fusion rules, fusion parameter selection relying on experience, and face fusion artifacts.

With the development of deep learning technology, however, the issues mentioned above could be addressed to a large extent. The latest fusion methods for general purposes (such as objects, scenes, etc.) have already applied deep neural networks [36,37,38,39].

For example, Li et al. [36] were for the first to use the well-known VGG19 structure as the depth feature extractor combined with the $L_{1}$ norm to achieve deep fusion. Later, the same team replaced VGG19 with ResNet50 as the depth feature extractor to fuse an infrared image and visible image, achieving a fusion result higher than that of VGG19 on a public dataset [37].

Li’s team further proposed the DenseFuse network [38], which has a self-coding structure to realize encoding, fusion, and reconstruction. Their team achieved the best fusion result at the time.

Later on, Ma et al. [39] applied Generative Adversarial Network (GAN) techniques to solve the problem of image fusion and proposed a method called FusionGAN. Experiments on public datasets demonstrated that their fusion results look like sharpened infrared images with clearly highlighted targets and abundant detailed information. But, the fusion outputs of this model contain obvious distortion when applied to hyperspectral face images.

To summarize, these deep learning-based fusion methods can effectively avoid problems such as manual design of fusion rules and selection of fusion parameters while yielding high fusion performance (i.e., high image quality).

Nonetheless, none of these methods were designed for the case of hyperspectral face recognition (mostly designed for scenes and objects). The fusion of spectral face images across several spectral bands is a challenging problem that requires special treatment. Apart from this, many prior research works concentrated on how to output a high-quality image (in terms of SNR, sharpness, SSIM, entropy, etc)—in other words, an image with a good visual effect. However, they usually neglected facial characteristics (i.e., features) pertaining to a specific individual. As a result, even though these methods can output fused faces of high image quality, they usually do not guarantee that informative facial features are also preserved, which is crucial to the goal of high recognition accuracy in the context of hyperspectral face recognition. Whether these deep learning-based image fusion methods can ultimately improve recognition performance needs to be tested.

Therefore, in this work, we make the first attempt to study the problem of hyperspectral face recognition with a deep learning approach. We propose a feedback-style dense residual network for hyperspectral face fusion to achieve high-quality fused images and improved face recognition rates at the same time.

## 3. Proposed Methodology

In this section, the proposed method of deep learning-based hyperspectral face fusion (HyperFace) is explained. We describe the network architecture, recognition-oriented composite loss, and recognition stage. It should be noted that although we use the two bands of NIR and visible light as the fusion inputs, the proposed hyperspectral fusion method and idea can also be easily extended to fuse a set of more bands (such as MWIR and LWIR) at the same time.

Traditionally, image fusion (including facial image fusion) is achieved by hand-crafted operator-based methods, in which two input images are processed by a manually designed operator to extract features. These features are then fused according to a certain rule, followed by a final stage of image reconstruction to produce the fused output image from the fused features. More recently, due to their higher robustness, deep learning-based methods have replaced traditional methods, where all steps of feature extraction, fusion, and reconstruction are automatically accomplished. However, both traditional and deep learning-based image fusion methods are not designed for the specific problem of hyperspectral face recognition, where not only the quality of the fused face matters but also the identity-pertaining features are crucial. In other words, both visual quality and recognition performance should be considered. This issue is addressed in this work by proposing a new composite loss function that ensures recognition performance by including an identity-pertaining loss term. Additionally, in order to ensure high performance in feature extraction, we introduce both local and global residual learning techniques, which alleviate issues such as gradient vanishing and local maximum since the multispectral face dataset is limited compared to the visible light dataset (another issue unique to the problem of hyperspectral face recognition). A feedback connection is also proposed for the purpose of higher-quality reconstruction of the hyperspectral face. Lastly, the idea of pre-fusion is also proposed to prevent the network from being biased toward a single band during training.

### 3.1. Overall Network Architecture

Consider a pair of facial images in visible light $I_{Vis}$ and infrared $I_{IR}$, and assume that they are affinely aligned. The alignment is achieved by affine transformations such as translation, rotation, scaling, and shear based on the fiducial points of the pupil centers and nose tip [18] (other alignment methods, such as projective or other non-rigid transformations, can also be applied, which may slightly affect recognition performance according to other study reports). Denote a hyperspectral facial image by $I_{F}$. In this work, all images are grayscale images. The visible and infrared images are the inputs to the network, and the fused image is its output.

**Figure 1:**
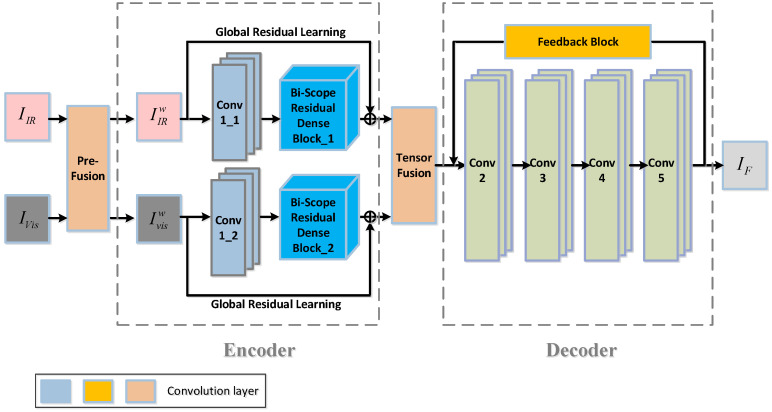
The overall architecture of the proposed network, HyperFace.

The proposed network consists of four modules: the pre-fusion layer, the encoder, the fusion layer, and the decoder. The overall architecture of the network is shown in Figure 1.

#### 3.1.1. The Pre-Fusion Scheme

Unlike other fusion models that directly fuse an input image pair in different bands, we introduce a pre-fusion layer at the beginning of our model. The purpose of the pre-fusion layer is to enable the network to make full use of the complementary information under different spectra. The reason previous methods may fail to utilize the complementary multispectral information of the face is that without such a pre-fusion layer, the training of the encoder starts with arbitrary values. Thus, network convergence is much more difficult, and a local optimum is more likely to happen. Therefore, using the pre-fusion layer prevents the network from being biased toward a single spectrum during training, thus avoiding an excessive emphasis on the characteristics of one spectrum.

As shown in Figure 1, at the front part, the pre-fusion layer takes in the original infrared ($I_{IR}$) and visible light ($I_{Vis}$) face images and blends one spectrum with the other spectrum. The outputs are weighted summations of the infrared face and the visible light face, resulting in two sets of pre-fused facial images, $I_{IR}^{w}$ and $I_{vis}^{w},$ which are then sent to the following encoder. Since different bands have an uncertain impact on the final fused face image, the blending ratio between the different bands needs to be considered. Therefore, we further introduce a weight for each band of IR and visible light. Mathematically, the outputs of the pre-fusion layer are described as

(1) $$\left\{\begin{array}{c} I_{IR}^{w}=a_{1}I_{IR}+a_{2}I_{vis}\;\\ I_{vis}^{w}=a_{2}I_{IR}+a_{1}I_{vis} \end{array}\right.$$

where $a_{1}+a_{2}=1$. The weights of $a_{1}$ and $a_{2}$, which are 0.8 and 0.2, respectively, are determined empirically during our experiments. From the empirically determined values of the weights, we can see that the pre-fusion strategy is indeed beneficial for preventing the network from being biased toward a specific band (otherwise, the weights would be a very small value).

The pre-fused outputs for the visible image ($I_{IR}^{w}$) and infrared image ($I_{vis}^{w}$) contain information from both spectra. This ensures that the subsequent training process is unbiased toward the encoding and reconstruction of the fusion image, rather than favoring a certain spectral image, which is conducive to improving the fusion effect.

#### 3.1.2. The Encoder Module

**Figure 2:**
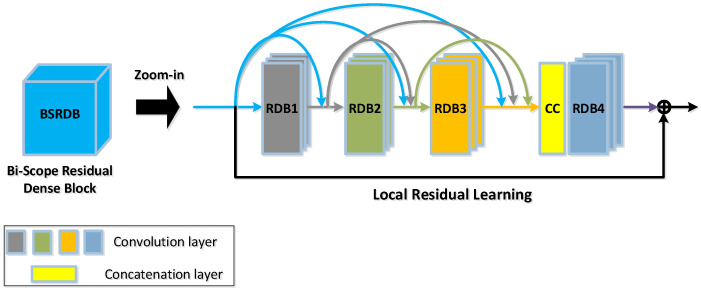
A zoomed-in view of the architecture of the bi-scope residual dense block (BSRDB).

Following the pre-fusion module is the encoder module, which is a Siamese-architecture sub-network (see the front part in Figure 2 for the overall architecture and Figure 2 for more details, where the bi-scope residual dense block is expanded to include detailed layers such as convolutional layers and concatenation layers). The encoder comprises two streams: the top stream is for the IR face and the bottom one is for the visible light face. Each stream consists of a convolutional layer (Conv1) and a cascaded connection of several residual dense blocks, which we term the Bi-Scope Residual Dense Block (BSRDB). The convolutional layer (denoted as Conv1_*x*, where x=1 stands for the IR and x=2 stands for visible light) is designed to extract rough features, while the bi-scope residual dense block (BSRDB_1 or BSRDB_2) is meant to extract higher-level and finer features. Both layers of Conv1_1 and Conv1_2 are designed with 1 input channel and 16 output channels using a convolutional kernel size of 3×3, and the ReLU function is used as the activation function.

As shown in Figure 2, the BSRDB consists of several building blocks called dense blocks (DBs) [40]. The DBs are essentially convolutional layers connected in a dense way such that the outputs of all previous convolutional layers are spliced at the next layer (see Figure 2). More specifically, the BSRDB in this work consists of three DBs followed by a concatenation layer, which concatenates all the features at the output of DB3. The concatenated features are then sent to the fourth DB. All the DBs use a convolutional kernel size of 3×3 with a ReLU activation function. For the first three DBs, all the output feature maps have 16 channels, while the numbers of the input channels increase by 16 due to the fact that the output channels of a previous DB are added to the input channels of the current DB. For DB4, both the input and output feature maps have 64 channels. In this way, a multi-dimensional feature map can be obtained.

Inspired by the works of He et al. [41] and Zhang et al. [42], we introduce the idea of *local* residual skip learning to enhance the dense blocks. This enables the extraction of abundant local facial features and further improves the network’s representation ability, resulting in better fusion performance. Moreover, using residual skip connections can effectively alleviate the issues of gradient vanishing and local maximum (since the multispectral face dataset is limited in size compared to the visible light dataset) during the training of the deep networks. Therefore, it improves the training efficiency of the networks. The connections of the local residual learning jump between the first DB and the last DB, denoted by the skip connection and the sum symbol, as shown in Figure 2.

In addition to local residual learning among the RDBs, we further add *global* residual learning to the entire encoder, which skips Conv1 and the BSRDB (refer to Figure 1 for details). Through this global residual connection, we can obtain maximal detailed information from the source image and the intermediate layer image, which is conducive to the full fusion of the image. Such a design of the BSRDB makes full use of the information from all local layers to improve the network’s representation ability. The weights of the two encoder streams are tied, i.e., Conv1_1 and Conv1_2, as well as BSRDB_1 and BSRDB_2, share the same weights.

#### 3.1.3. The Fusion Strategy

At the output of the encoder module, the IR and visible light faces are represented as multi-dimensional feature maps, which need to be further fused. Determining how to fuse the feature maps needs a specific fusion rule. Previous research works (especially traditional hand-crafted operator-based methods) have utilized different fusion rules, such as the maximum rule, minimum rule, summation/average rule, and other adaptive rules [43]. Considering that the parameters of our network are learnable (meaning that the fusion strategy is automatically optimized by the training process) and in order to keep our network succinct without unnecessary redundancy, we choose a summation-based fusion strategy at the fusion layer.

**Figure 3:**
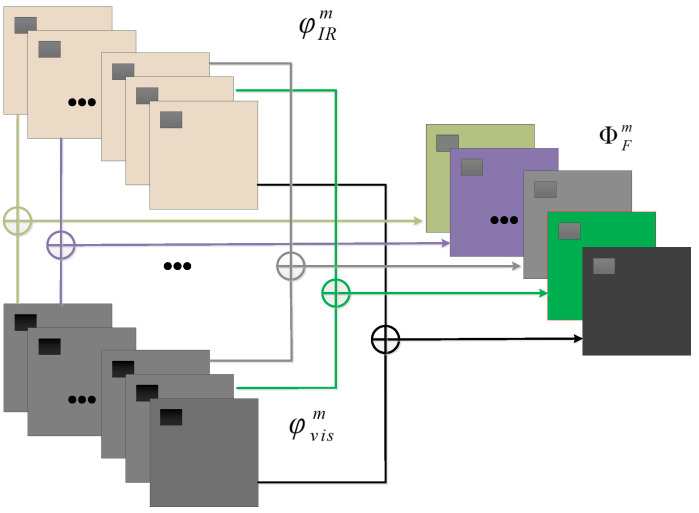
The fusion strategy: A fused feature map is the summation of an IR and a visible light feature map.

Once the encoder and decoder networks are trained, during the testing process, we use the encoder of a double-stream architecture whose weights are tied between the two streams. We choose a summation rule to combine the pairs of salient feature maps obtained by the encoder from both streams, i.e., the IR stream and the visible stream, resulting in the fused (i.e., hyperspectral) feature maps. It should be noted that the fusion module fuses the IR and visible light feature maps in the corresponding dimensions and at the corresponding pixel positions. The fusion strategy is illustrated in Figure 3.

Let $\varphi_{IR}^{m}\left(x,y\right)$ and $\varphi_{vis}^{m}\left(x,y\right)$, (m=1,2,...,M) be the feature maps obtained by the encoder from the input faces of IR and visible light, respectively, and let $\Phi_{F}^{m}\left(x,y\right)$ be the fused feature maps. Then, the fusion strategy can be formulated as

(2) $$\Phi_{F}^{m}\left(x,y\right)=\varphi_{IR}^{m}\left(x,y\right)+\varphi_{vis}^{m}\left(x,y\right),$$

where (x,y) is the pixel location in the input feature maps and fused feature maps. The output of $\Phi_{F}^{m}\left(x,y\right)$ then serves as the input for the following decoder to eventually reconstruct the fused hyperspectral facial image.

#### 3.1.4. The Feedback-Style Decoder

Finally, a decoder module is designed to reconstruct the hyperspectral face from the high-level features at the fusion layer. We introduce a feedback-style decoder to boost our model, rather than using a simple uni-directional decoder as in many other deep learning research works. More specifically, a feedback block is designed to add feedback connections that generate powerful high-level representations. The feedback mechanism allows the decoder network to carry a notion of the output such that the previous states can be rectified. In our network, the introduction of this feedback block corrects the input facial features of the decoder with the output, resulting in a reconstructed facial image that is more realistic and of higher quality.

**Figure 4:**
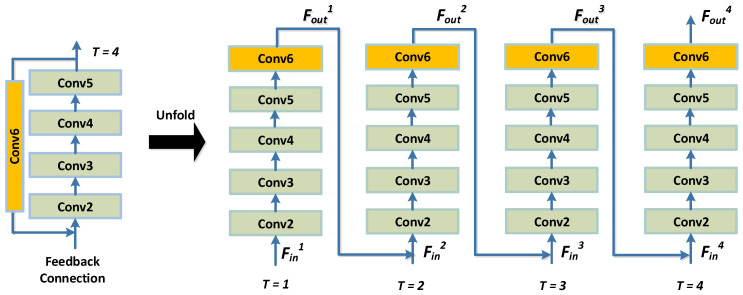
The decoder with the feedback connections unfolded.

Particularly, the feedback decoder comprises five convolutional layers (denoted as Conv2–Conv5) and a feedback block (FB). The five convolutional layers constitute the main body of the decoder and are responsible for a rough reconstruction of the fused image (i.e., the hyperspectral face). All five layers use the ReLU function as the activation function. The feedback block is then connected from the output of the decoder to the input, which is responsible for rectifying and refining the final reconstructed image. The FB is essentially a convolutional layer but has no activation function. The purpose of such a structure is to ensure the linear transmission of information during the feedback process. The unfolded structure of the feedback decoder is shown in Figure 4.

As shown in Figure 4, the feedback connection at the *t*-th iteration receives the feedback information from the previous output, $F_{out}^{t-1}$, to correct the feature representation extracted from the encoder, $F_{in}^{t}$, and then passes a more powerful representation, $F_{out}^{t}$, to the next iteration and the reconstruction block. At the very beginning of the feedback decoder, $F_{in}^{t}$ and $F_{out}^{t-1}$ are concatenated and compressed by four convolution operations, Conv(3,m) with ReLU activation, where *m* denotes the base number of filters. Then, $F_{out}^{t}$ is obtained by a convolution operation (denoted by Conv6) without any activation function. The number of feedback connections used in this paper is empirically chosen as 4.

The above iteration process at time point *t* can be mathematically formulated as

(3) $$F_{out}^{t}=Conv\left[Conv{\left(F_{in}^{t},F_{out}^{t-1}\right)}_{relu}^{4}\right]$$

where Conv(·) stands for a convolution operation. $F_{in}^{t}$ and $F_{in}^{t-1}$ are the inputs at time *t* and t−1, respectively. $F_{out}^{t}$ is the reconstructed image.

To summarize the structure of the four modules, we list all the specific parameters of the proposed network in Table 1.

### 3.2. Recognition-Oriented Composite Loss

After designing the hyperspectral fusion network, the next critical step is to choose an effective loss function. Current works on image fusion usually overlook or downplay the importance of this problem. Either a standard basic loss function is used or a more sophisticated loss function is selected, but it is not adjusted to the ultimate task of face recognition. In view of this, we propose a composite recognition-oriented loss function, which is driven by both the fusion image’s quality and facial details.

**Figure 5:**
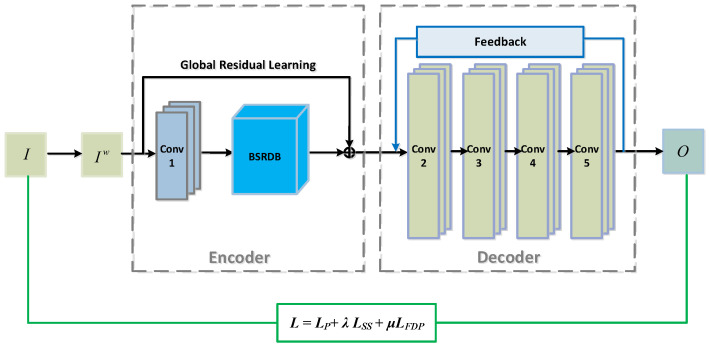
The framework of the recognition-oriented composite loss during training.

In order to guarantee the high quality of the fused image, which results in good recognition performance, we need to reconstruct the input fusion image as precisely as possible, and at the same time, preserve as many facial details as possible to enhance recognition performance. Therefore, we design a composite loss function *L* to train our encoder and decoder,

(4) $$L=L_{P}+\lambda L_{SS}+\mu L_{FDP}$$

which is a weighted combination of the pixel loss $L_{P}$, structural similarity loss $L_{SS}$ with weight λ, and average gradient loss $L_{FDP}$ with weight μ. Throughout the experiments, the weights of λ and μ are empirically determined to be 0.69 and 0.05, respectively. The recognition-oriented composite loss function and the corresponding training process are shown in Figure 5.

#### 3.2.1. Pixel Loss

The first component of the total composite loss is the pixel loss, $L_{p}$, between the input and output of the overall network, as shown in Figure 5. This pixel loss provides a basis for convergence between the input and the output of the network. In other words, given an input face of any light band, we seek to train the network so that the reconstructed output of the decoder is as close as possible to the input. The pixel loss, $L_{p}$, is calculated as

(5) $$L_{P}={∥O-I∥}_{2}$$

where *O* and *I* indicate the output and input face images, respectively. It is the L2 norm (i.e., Euclidean distance) between the output, *O*, and the input, *I*.

#### 3.2.2. Structural Similarity Loss

We select the structural similarity loss as the second component of the composite loss function. The reason we include this component is that the pixel loss alone cannot ensure good reconstruction quality. In certain cases, a very small average pixel difference could lead to dramatic changes in visual perceptual quality, such as when blurring and salt-and-pepper noise are present. In these cases, structural similarity should be better considered between the reference and distorted images [44].

Therefore, we introduce the structural similarity loss, $L_{SS}$, and we add it to the pixel loss. $L_{SS}$ is mathematically described as

(6) $$L_{SS}=1-\frac{\left(2\mu_{I}\mu_{O}+C_{1}\right)\left(2\sigma_{IO}+C_{2}\right)}{\left(\mu_{I}^{2}+\mu_{O}^{2}+C_{1}\right)\left(\sigma_{I}^{2}+\sigma_{O}^{2}+C_{2}\right)}$$

where $\mu_{I}$ and $\mu_{O}$ are the mean intensity of *I* and *O*, respectively, and $\sigma_{I}$, $\sigma_{O}$, and $\sigma_{IO}$ are the standard deviation of *I*, the standard deviation of *O*, and the covariance of *I* and *O*, respectively. $C_{1}$ and $C_{2}$ are two small constants included to ensure stability when the two factors of the denominator, i.e., $\mu_{I}^{2}+\mu_{O}^{2}$ and $\sigma_{I}^{2}+\sigma_{O}^{2}$, are close to zero.

#### 3.2.3. Facial Detail Preserving Loss

Finally, we introduce a loss component that preserves facial details, which are crucial for successful face recognition operations. This loss component overcomes the issue with many other image reconstruction algorithms where only the image quality of the reconstructed image is considered, while any specific applications following image reconstruction are ignored. In other words, our design of the facial detail-preserving loss is face recognition-oriented, which ensures both high-quality reconstruction and high-performance recognition. $L_{FDP}$ is defined as

(7) $$L_{FDP}=\frac{1}{M\times N}\sum_{i=1}^{M}\sum_{j=1}^{N}\sqrt{\frac{{\left(\frac{\partial O}{\partial x}\right)}^{2}+{\left(\frac{\partial O}{\partial y}\right)}^{2}}{2}}$$

where M×N represents the size of the image, while $\frac{\partial O}{\partial x}$ and $\frac{\partial O}{\partial y}$ denote the gradients of the output image, *O*, in the horizontal and vertical directions, respectively. It is worth noting that unlike the pixel loss and structural similarity loss, the facial detail-preserving loss is reference-free, meaning that no reference image is required during its calculation. Such a design is very convenient for use. Moreover, it could be used in other applications and is not limited to image fusion.

### 3.3. The Recognition Stage

**Figure 6:**
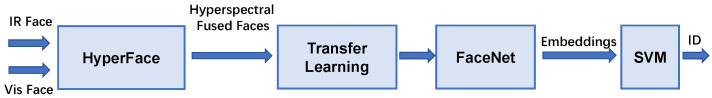
The pipeline of the full process of hyperspectral face recognition.

After the fusion of IR and visible light facial images by the fusion network with the composite loss, the problem of hyperspectral face recognition is now simply reduced to a problem of intra-spectral face recognition, in other words, matching fused facial images. One could simply apply the very recent accomplishments of deep learning to this problem. However, this idea suffers from the problem of a small amount of data, as coupled IR and visible light data are difficult to obtain. Therefore, we use the transfer learning technique to train the well-known deep learning model of face recognition, FaceNet [45]. More specifically, FaceNet is pre-trained on the LFW dataset [46] and then fine-tuned using the aforementioned multispectral datasets. Such a transfer learning technique is theoretically feasible because IR faces show a certain level of similarity to visible faces, especially for the near-infrared band (which is the case in this paper), e.g., the edges of facial parts are similar. Thus, a large part of the low-level features of IR and visible faces would be shared for the FaceNet model during transfer learning, which would undoubtedly lower the complexity of transfer learning. Nonetheless, FaceNet was trained to classify a different dataset of different identities than our datasets. Further, we combine the output embeddings of the transfer-learned model with a support vector machine (SVM). The diagram of the full process of hyperspectral face recognition is given in Figure 6.

#### 3.3.1. Transfer Learning

Transfer learning is a technique of transferring information from a related domain to improve algorithm performance in another domain [47]. The purpose of transfer learning is to extract knowledge from one or more source tasks and apply that knowledge to a target task. Therefore, transfer learning technology can transfer knowledge from recognition algorithms or network models of high performance that are trained in a relevant Source Domain (SD) so that they can achieve boosted performance in the Target Domain (TD) with a small amount of data.

In this paper, the FaceNet model trained on the LFW dataset was chosen as the basis to obtain the entire transfer model and its hyperparameters, and then the CASIA and Q-FIRE datasets were used to train the transferred model. It should be noted that we chose FaceNet only because it has been widely accepted as a deep learning-based baseline model. In practice, it can be easily replaced with any other successful model following the overall pipeline of hyperspectral face recognition.

#### 3.3.2. FaceNet

FaceNet is an end-to-end deep learning model for face recognition. It directly learns a mapping from a facial image to a compact Euclidean space where the distance directly corresponds to a measure of face similarity. Once this space has been produced, tasks such as face identification, verification, and clustering can be easily implemented using standard techniques treating FaceNet embeddings as feature vectors.

In our paper, the FaceNet model is used to train a 128-dimensional feature vector corresponding to each face image, and SVM is sequentially used to classify and recognize these 128-dimensional features. It should be noted that other face recognition models, such as DeepFace and ArcFace, can also be used as the face recognition model after the hyperspectral face fusion stage. The overall idea is similar. During the experiments, the classification accuracy is evaluated in order to compare the efficiency of different hyperspectral fusion methods.

## 4. Experimental Setup

### 4.1. Datasets

In our experiments, we use two datasets: the Quality-Face/Iris Research Ensemble (Q-FIRE) dataset collected by Clarkson University [48] and the CASIA NIR-VIS 2.0 Face Database (CASIA) acquired by the Institute of Automation, Chinese Academy of Sciences [49]. It should be noted that there are currently much fewer multispectral face datasets (i.e., datasets containing visible light and IR faces for the same subject) publicly available than public datasets for visible light face recognition. To make matters worse, these public multispectral face datasets are mostly quite limited in size compared to the visible light face datasets. Evaluating the performance of the proposed methodology with a trustworthy conclusion remains a challenge. To address this issue, we choose three well-known multispectral face datasets (rather than just a single dataset) and evaluate our method on all three datasets. Furthermore, techniques such as transfer learning and data augmentation are all involved on top of the design of a highly efficient network structure, which is described in detail in Section 3.3 and Section 4.2.

The Q-FIRE dataset comprises color and NIR face images of 82 subjects whose ethnicities include Caucasian, African American, and Asian. Both female and male subjects are present, and their ages vary from young to elderly. Each class is represented by two or four color and NIR images collected during two different visits. All face images have a neutral expression. Different demographic factors such as gender, age, and race are accounted for. The quality of the collected images is relatively high. All visible light images have a resolution of 1920×1080, while the resolution of the NIR images is 2352×1728. All color images are saved in .bmp format, while all NIR images are saved in .png format. The total number of images in CASIA is 2063.

The CASIA dataset is composed of four subsets, each of which was collected at a different time. The dataset contains face images from 725 Asian individuals of different ages and genders. Each session comprises face images in two light bands, i.e., visible and NIR face images. The image quality of the dataset is not very high. All face images have a resolution of 480×640 and are saved in .bmp format. The total number of images in CASIA is 17580.

The TINDERS dataset is composed of 96 frontal face classes (Caucasian/African American and female/male), each represented by visible and NIR (980 nm) at three different standoff distances (1.5 m, 50 m, and 106 m). At each distance and spectrum, four or five images per class are available: two or three with a neutral expression and two with a talking expression. The resolution of the NIR face images is 640×512 (.png format). The visible (color) images, with a resolution of 480×640 (.jpg format), were collected at a short distance and in two sessions (three images per session), all with a neutral expression. The total number of images in TINDERS is 2590.

Face images from all three datasets are aligned and cropped beforehand using the coordinates of the eyes. It is worth mentioning that although the original resolution of the images in the three datasets varies, we normalize all images to the same size (160×160). This is done to ensure a fair comparison. Before hyperspectral fusion, all color facial images are converted to grayscale images. This is done by retaining the luminance channel while eliminating the hue and saturation channels in the LAB color space: $I_{gray}=0.2989\times R+0.5870\times G+0.1140\times B$, where *R*, *G*, and *B* are the three channels of an input color image and $I_{gray}$ is the converted grayscale output. After alignment, cropping, and grayscale conversion, the pixel values of all the IR and visible light images are normalized to within the range of [0, 1].

**Figure 7:**
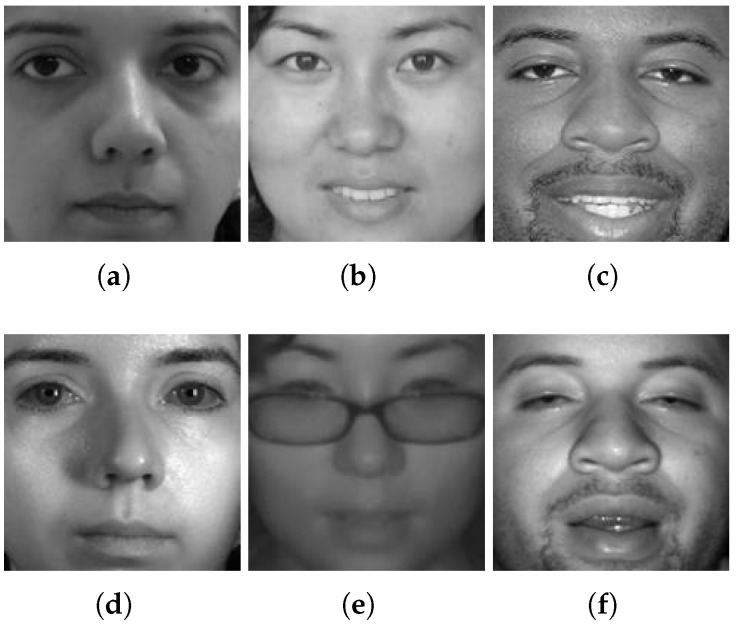
Pairs of sample multispectral faces from three different datasets: Q-FIRE (left column), CASIA (central column), and TINDERS (right column). (**a**–**c**) in the top row show the visible light faces, while (**d**–**f**) in the bottom row are the corresponding visible light faces.

During the experiments, the proposed network is trained and tested with a ratio of 3:1. Pairs of sample facial images from the three datasets are shown in Figure 7.

### 4.2. Training

The aim of the training process is to train the encoder and decoder of HyperFace so that they achieve optimal feature extraction and reconstruction. It should be noted that during training, the fusion layer does not need to be involved. In other words, training faces of IR and visible light go through the pre-fusion, encoder, and decoder modules (refer to Figure 5). The fusion layer is only needed when a hyperspectral fused face is generated in the testing process. During testing, a pair of IR and visible light faces is initially decomposed by the encoder, then fused by the fusion layer using the feature maps, and finally reconstructed by the decoder to generate a fused hyperspectral face.

The network is trained using the infrared and visible images from CASIA and Q-FIRE. The datasets are divided into training and testing data following a ratio of 3:1. All of the training data are resized to 256×256. The RGB images are transformed into grayscale images. During training, the Adaptive Moment Estimation (Adam) optimizer is used, and the initial learning rate is set as η=0.0001. The batch size and epochs are 32 and 200, respectively. In order to mitigate the limitations of image size, data augmentation techniques such as translation, rotation, flipping, cropping, and jittering are utilized. Our method is implemented on a workstation with a Core i9 CPU@3.6 GHz, 16GB of RAM, and a GTX 2080Ti GPU.

**Figure 8:**
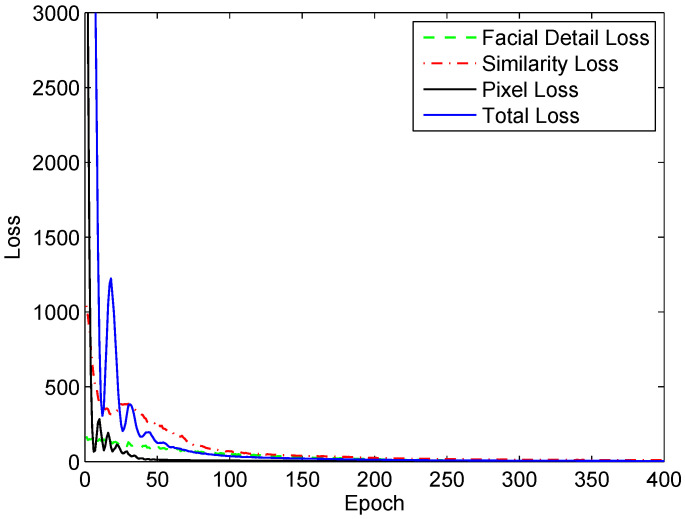
The plot of the training in terms of the composite loss and its components versus the number of epochs.

We generate the loss plot of the training process for the CASIA dataset, in which the total loss and its components—the pixel loss, SSIM loss, and facial detail loss—are compared (as shown in Figure 8). Due to the fact that the three components differ in their orders of magnitude, we normalize them to the same range for the purpose of better visualization.

## 5. Experimental Results and Analysis

In this section, we first discuss the training process and then use several different types of image quality metrics to evaluate the proposed fusion method and compare it with other methods. Lastly, we carry out experiments of hyperspectral face recognition to further demonstrate the superiority of the proposed method in terms of the recognition rate.

### 5.1. Fusion Performance Evaluation

In order to justify our proposed model, we compared HyperFace with four other fusion methods and calculated the quality metrics of the corresponding fused faces. The four fusion methods were chosen in a representative way and included the three best traditional/non-deep learning methods, as well as a deep learning-based method. Specifically, they are the wavelet transform (WT) fusion method [34], the cross-bilateral filter (CBF) fusion method [50], the joint-spare representation (JSR) method [51], and the deep learning-based method DenseFuse [38].

**Figure 9:**
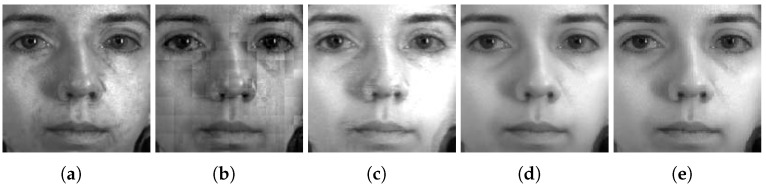
Comparison of different fusion methods on the Q-FIRE dataset. From left to right are the results of: (**a**) CBF [50], (**b**) WT [34], (**c**) JSR [51], (**d**) DenseFuse [38], and (**e**) the proposed method.

**Figure 10:**
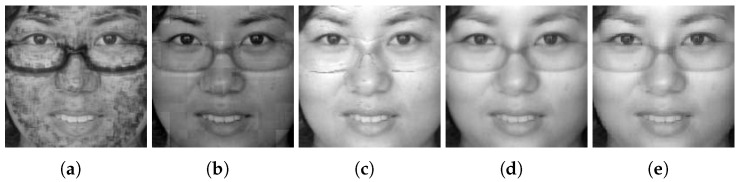
The results of different fusion methods on the CASIA dataset. From left to right are the results of: (**a**) CBF [50], (**b**) WT [34], (**c**) JSR [51], (**d**) DenseFuse [38], and (**e**) the proposed method.

**Figure 11:**
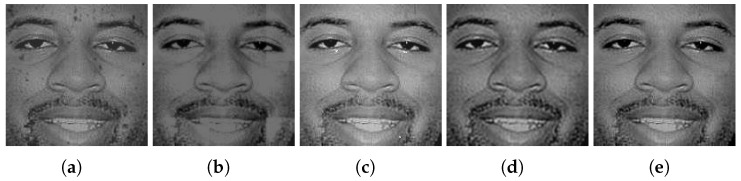
The results of different fusion methods on the TINDERS dataset. From left to right are the results of: (**a**) CBF [50], (**b**) WT [34], (**c**) JSR [51], (**d**) DenseFuse [38], and (**e**) the proposed method.

From Figure 9, Figure 10 and Figure 11, we can see that the fused hyperspectral faces contain complementary components from both the visible light band and the IR band. However, the visible light component seems to be more pronounced. Thus, we can state that the visible light band is more important for retaining high recognition performance, while the IR band is also beneficial for improving recognition performance. This observation can be attributed to the fact that the visible light band yields high-quality face images that contain informative facial features such as the edges and contours, and the IR band is invariant to lighting changes, providing complementary information to the visible light band.

For the purpose of quantitative comparison between our fusion method and the other four methods, we chose a total of six different types of widely used image quality metrics to evaluate the fusion performance. These metrics are entropy (EN) [52], image edge fidelity ($Q_{abf}$) [53], modified structural similarity for no-reference image (SSIM) [54], a non-reference image fusion metric (FMI) [55], peak signal-to-noise ratio (PSNR), and the Tenengrad sharpness. For all of these metrics, a higher value indicates higher fusion performance. It is worth noting that these metrics each have quite different natures and thus can evaluate the fusion performance in a comprehensive way.

An example of fused hyperspectral faces using the five fusion methods is shown side by side in Figure 9, Figure 10 and Figure 11 for Q-FIRE, CASIA, and TINDERS, respectively. As seen from Figure 9a,b, when tested on Q-FIRE, the fusion results of both CBF and WT contain apparent artifacts, with the former displaying dot-shaped artifacts and the latter block-shaped artifacts. The fusion result of JSR is visually better in quality but still demonstrates some white specks around the ocular and nasal areas, as well as a strip shade around the left side of the chin. Compared with CBF, WT, and JSR, the deep learning-based method DenseFuse yields a significantly better fusion result, but it is still noticeably blurry. When fusing with HyperFace (our proposed method), we can see that the overall visual quality is the best. When the same test was conducted on CASIA (see Figure 10) and TINDERS (see Figure 11), we again see that DenseFuse and HyperFace are more advantageous over the three traditional methods, with HyperFace being the best. The artifacts of CBF in this case are even more pronounced, which suggests that CBF is not suitable for face fusion, even though it is successful at dealing with general-purpose image fusion.

To objectively and quantitatively evaluate the fusion performance, we determined the image quality using the six aforementioned metrics. The average values of the metrics for all fused images in the three datasets are listed in Table 2, Table 3 and Table 4. The best values of the five different fusion methods are indicated in bold. When the test dataset was set as Q-FIRE, the proposed method, HyperFace, achieved the best values in four out of the six metrics: $Q_{abf}$, SSIM, FMI, PSNR, and Tenengrad. For the other two metrics, EN and PSNR, HyperFace achieved the second-best values (shown in cyan). This result indicates that our method can preserve more structural details, introduce less artificial noise during fusion, and retain more useful features and information than the other methods.

Similarly, when CASIA was tested, HyperFace achieved the best values in five out of the six metrics: EN, $Q_{abf}$, SSIM, PSNR, and Tenengrad, with the FMI value being the second best. Lastly, when TINDERS was tested, HyperFace once again achieved the best values across the six metrics. Overall, we can conclude that our proposed method performs better than the other fusion methods in terms of fusion quality.

### 5.2. Recognition Performance Evaluation

We further conducted experiments to analyze the efficiency of the proposed hyperspectral face fusion from the perspective of face recognition accuracy. Such a perspective is crucial because good performance in terms of image fusion quality does not guarantee a high recognition rate, which is a shortcoming of many general-purpose fusion methods. Therefore, we wanted to ensure that our method demonstrates high performance in terms of both fusion quality and recognition accuracy.

We first used the five fusion methods described in the previous subsection to obtain five groups of hyperspectral fused faces. Then, we used FaceNet [45] and support vector machine (SVM) to identify the fused faces for the five fusion methods. The recognition results are listed in Table 5.

In order to demonstrate that hyperspectral fusion is advantageous over single-spectrum-based face recognition (i.e., using either IR or visible light), we compared the recognition accuracy of the five fusion methods with the recognition accuracy when only IR or visible light data are used.

As seen in Table 5, when tested on Q-FIRE, the single-spectral face recognition rates were relatively lower, with visible light and IR being 97.64% and 92.57%, respectively. The fact that the recognition rate of the visible light was higher than that of IR was expected, considering that IR images are usually of lower quality. When face recognition was conducted in a hyperspectral manner (i.e., after fusing IR and visible light), all the fusion methods displayed improved recognition performance compared to the single-spectral IR case, but the three traditional methods behaved worse in hyperspectral face recognition than in visible light face recognition. Nonetheless, both deep learning methods (i.e., DenseFuse and our proposed model, HyperFace) in the hyperspectral case all surpassed the visible light case. This justifies the topic of hyperspectral face recognition that we study in this paper.

We conclude that among the five fusion methods, the two deep learning-based methods (DenseFuse and HyperFace) achieved significantly higher performance compared to the three traditional methods, CBF, WT, and JST. Our proposed method, HyperFace, demonstrates higher performance than DenseFuse, achieving a recognition rate of 99.25% on the Q-FIRE dataset. These results show that the proposed method not only improves the image quality of fused hyperspectral faces but also boosts the accuracy of face recognition.

When the test dataset was set as CASIA, as shown in Table 5, we once again observed a similar performance trend: the proposed method was the best in terms of the recognition rate, although all five methods yielded lower recognition rates on the CASIA dataset than on the Q-FIRE dataset (which was expected since the latter dataset has higher image quality and a smaller number of subjects). Face recognition in visible light was better than that in IR, and hyperspectral face recognition was better than face recognition within a single spectrum, either visible light or IR. Finally, when the last dataset TINDERS was tested (see Table 5), a similar trend was observed: the face recognition rate in visible light was higher than that in IR, and hyperspectral face recognition using image fusion was better than face recognition using a single band of visible light or IR. Once more, our fusion method (HyperFace) performed better than all other fusion methods in terms of both the recognition rate and image quality (see Table 4).

In order to analyze the computational efficiency of the proposed method, HyperFace, and the other methods involved (which is especially important from the perspective of real-world deployment), we further carried out experiments on the computation time to compare the different methods. Both the hyperspectral fusion time of a pair of IR and visible light faces and the subsequent recognition time using the FaceNet model were measured and recorded in seconds. Note that the time was averaged across all three datasets. A summary of the computation times is given in Table 6. As seen from the results, our proposed method was among the top performers—only 0.003 s slower than the fastest method, DenseFuse. Both DenseFuse and our proposed method achieved an FPS of more than 20, which meets the requirement for real-time processing.

After analyzing all the presented experimental results, we conclude the following:

- Our proposed method, HyperFace, achieves the highest image fusion quality compared to the other four fusion methods in terms of six different types of image quality metrics.
- The recognition rate of our proposed method is also the highest compared with the other four fusion methods. The proposed method can work in real time.
- Hyperspectral face recognition by fusing IR and visible light faces is more advantageous than single-band face recognition.

## 6. Conclusions and Future Work

In this research work, we introduced deep learning as a solution to the problem of hyperspectral face recognition and formulated a complete framework, the core of which is a newly designed deep fusion model (HyperFace). Our proposed model comprises four modules: a pre-fusion module, an encoder, a fusion module, and a decoder. First, the pre-fusion module takes in the source faces in both IR and visible light to make full use of multispectral facial information during training. Then, the face features are decomposed and extracted by the encoder, which is characterized by several bi-scope residual dense blocks with both local and global residual learning connections. Thereafter, the fusion module fuses and integrates the feature maps of IR and visible light data into one set of feature maps, which contains all salient features from the source images. Finally, a fused image (i.e., the hyperspectral face) is reconstructed by a feedback-style decoder. A composite loss, which is recognition-oriented and preserves useful facial details, is designed to boost performance.

We use different types of image quality metrics to evaluate the image fusion performance of our model. Experiments demonstrate that our deep model outperforms other methods, which are either traditional or deep learning-based, on both the CASIA and Q-FIRE datasets. To further justify the model, we also test the face recognition accuracy. Again, the proposed method outperforms all the other methods.

Although the proposed methodology is verified on public datasets and proven to exhibit satisfactory performance, additional real-life and practical scenarios need to be tested. Current public datasets are usually collected in indoor setups in the laboratory. The “nighttime” condition is usually simulated by turning off any visible light lamps so the room is dark. Future work needs to be carried out to collect and evaluate real-life scenario datasets (especially on multispectral datasets of a larger size under uncontrolled conditions with variations in facial expression, pose, lighting, etc.).

In addition to the near-IR (NIR) band considered in this work, other IR bands, such as short-wave IR (SWIR), mid-wave IR (MWIR), and long-wave IR (LWIR), also need to be tested and evaluated in future work. Moreover, since IR face images are usually of lower quality compared to visible light face images due to limitations in its imaging mechanism, image enhancement prior to hyperspectral face fusion could be a potential solution to avoid the phenomenon of imbalance in heterogeneous quality. Lastly, other types of image fusion strategies can also be adapted for the application of hyperspectral face recognition, such as Generative Adversarial Network (GAN)-based methods, reinforcement learning-based methods, etc. Domain adaptation-based techniques are also potential research directions that provide an alternative approach to transfer learning, as utilized in this work, considering that current IR imagery datasets are quite limited in size.

## Figures and Tables

**Table 1:** Summary of parameters of the proposed network. Conv denotes the convolutional layer and CC stands for concatenation.

Block Name	Layer	Kernel Size	Input Channels	Output Channels	Activation
Encoder	Conv1	3	1	16	ReLU
	BSRDB	3	16	64	ReLU
Decoder	Conv2	3	64	64	ReLU
	Conv3	3	64	32	ReLU
	Conv4	3	32	16	ReLU
	Conv5	3	16	1	-
	FB	3	1	64	-
Bi-Scope Residual Dense Block (BSRDB)	DB1	3	16	16	ReLU
	DB2	3	32	16	ReLU
	DB3	3	48	16	ReLU
	CC	3	64	64	-
	DB4	3	64	64	-
Feedback					
(FB)	Conv6	3	1	64	-

**Table 2:** The values of different types of quality metrics on the Q-FIRE dataset for different fusion methods.

Method	EN	$Q_{abf}$	SSIM	FMI	PSNR	Tenengrad
CBF [50]	**7.793**	0.2005	0.5078	0.7846	17.32	0.7210
WT [34]	7.418	0.1904	0.5127	0.8376	15.08	0.7414
JSR [51]	7.706	0.2075	0.5854	0.8518	19.35	0.7369
DenseFuse [38]	7.696	0.4963	0.9224	0.8915	**21.35**	0.7521
**Proposed**	7.752	**0.5463**	**0.9386**	**0.9078**	21.03	**0.8320**

The best values of different quality metrics are shown in bold.

**Table 3:** The values of different types of quality metrics on the CASIA dataset for different fusion methods.

Method	EN	$Q_{abf}$	SSIM	FMI	PSNR	Tenengrad
CBF [50]	6.758	0.2500	0.7001	0.7092	17.32	0.4363
WT [34]	6.593	0.3040	0.8238	0.8577	15.87	0.4835
JSR [51]	7.072	0.2990	0.8187	0.7856	20.78	0.4453
DenseFuse [38]	7.200	0.5913	0.9509	**0.8930**	23.02	0.4208
**Proposed**	**7.202**	**0.5913**	**0.9514**	0.8927	**23.06**	**0.5218**

The best values of different quality metrics are shown in bold.

**Table 4:** The values of different types of quality metrics on the TINDERS dataset for different fusion methods.

Method	EN	$Q_{abf}$	SSIM	FMI	PSNR	Tenengrad
CBF [50]	7.135	0.2013	0.6765	0.7569	17.48	0.6961
WT [34]	6.857	0.1982	0.5106	0.7427	15.72	0.7011
JSR [51]	7.108	0.3487	0.5043	0.8133	19.66	0.7138
DenseFuse [38]	7.226	0.5635	0.8576	0.8741	21.87	0.7164
**Proposed**	**7.327**	**0.5782**	**0.8612**	**0.8765**	**21.98**	**0.7491**

The best values of different quality metrics are shown in bold.

**Table 5:** Comparison of recognition rates when applied to Q-FIRE, CASIA, and TINDERS. The first two rows correspond to face recognition in a single spectrum, both IR and visible light, i.e., without hyperspectral fusion; the next three correspond to hyperspectral fusion using non-deep learning methods; and the last two correspond to the two deep learning-based approaches.

Method	Recognition Rate (%)		
	Q-FIRE	CASIA	TINDERS
Visible face	97.64	96.50	97.13
Infrared face	92.57	91.33	92.10
CBF [50]	92.81	91.07	92.55
WT [34]	94.34	92.58	93.46
JSR [51]	95.79	94.38	95.47
DenseFuse [38]	98.48	96.43	97.68
**Proposed**	**99.25**	**98.58**	**99.04**

The best value of different recognition methods on each dataset is shown in bold.

**Table 6:** Comparison of computation time between different methods. The total time is the summation of the hyperspectral fusion time and the recognition time. All time is measured in seconds (s).

Method	Hyperspectral Fusion Time (s)	Recognition Time (s)	Total Time (s)
CBF [50]	0.063	0.027	0.090
WT [34]	0.049	0.027	0.076
JSR [51]	0.031	0.027	0.058
DenseFuse [38]	0.017	0.027	0.044
Proposed	0.020	0.027	0.047

## Data Availability

The original data of the CASIA multispectral facial dataset presented in this study are openly available on the website of CASIA at http://www.cbsr.ia.ac.cn/english/NIR-VIS-2.0-Database.html (accessed on 23 April 2024), subject to application and approval. As for the Q-FIRE dataset, the original data were obtained from Clarkson University and are available from Prof. Stephanie Schuckers with proper permission. To access the TINDERS dataset, contact Dr. Brian Lemoff from the West Virginia High Technology Consortium Foundation to obtain permission.
